# Characterization of a TatA/TatB binding site on the TatC component of the *Escherichia coli* twin arginine translocase

**DOI:** 10.1099/mic.0.001298

**Published:** 2023-02-15

**Authors:** Emmanuele Severi, Mariana Bunoro Batista, Adelie Lannoy, Phillip J. Stansfeld, Tracy Palmer

**Affiliations:** ^1^​ Microbes in Health and Disease Theme, Newcastle University Biosciences Institute, Newcastle University, Newcastle upon Tyne, NE2 4HH, UK; ^2^​ School of Life Sciences and Department of Chemistry, Gibbet Hill Campus, University of Warwick, Coventry, CV4 7AL, UK

**Keywords:** mutagenesis, MD simulations, protein transport, TatC, Tat pathway, twin arginine signal peptide

## Abstract

The twin arginine transport (Tat) pathway exports folded proteins across the cytoplasmic membranes of prokaryotes and the thylakoid membranes of chloroplasts. In *Escherichia coli* and other Gram-negative bacteria, the Tat machinery comprises TatA, TatB and TatC components. A Tat receptor complex, formed from all three proteins, binds Tat substrates, which triggers receptor organization and recruitment of further TatA molecules to form the active Tat translocon. The polytopic membrane protein TatC forms the core of the Tat receptor and harbours two binding sites for the sequence-related TatA and TatB proteins. A ‘polar’ cluster binding site, formed by TatC transmembrane helices (TMH) 5 and 6 is occupied by TatB in the resting receptor and exchanges for TatA during receptor activation. The second binding site, lying further along TMH6, is occupied by TatA in the resting state, but its functional relevance is unclear. Here we have probed the role of this second binding site through a programme of random and targeted mutagenesis. Characterization of three stably produced TatC variants, P221R, M222R and L225P, each of which is inactive for protein transport, demonstrated that the substitutions did not affect assembly of the Tat receptor. Moreover, the substitutions that we analysed did not abolish TatA or TatB binding to either binding site. Using targeted mutagenesis we introduced bulky substitutions into the TatA binding site. Molecular dynamics simulations and crosslinking analysis indicated that TatA binding at this site was substantially reduced by these amino acid changes, but TatC retained function. While it is not clear whether TatA binding at the TMH6 site is essential for Tat activity, the isolation of inactivating substitutions indicates that this region of the protein has a critical function.

## Introduction

The general secretory (Sec) and twin arginine translocase (Tat) systems operate in parallel to export proteins across the cytoplasmic membranes of prokaryotes and the thylakoid membranes of plant chloroplasts [1, 2]. While the Sec pathway transports unfolded proteins, substrates of the Tat pathway are exported in a folded state [3, 4]. Proteins are targeted to the Sec or Tat machineries by the presence of a signal peptide at their N-terminus, which is usually cleaved during transport [5]. Sec and Tat signal peptides are superficially similar, but each has features that ensure passenger proteins are targeted to the appropriate transport system [6–8]. In particular, Tat signal peptides contain an almost invariant twin arginine motif that is critical for recognition by the Tat machinery [7, 9].

**Fig. 1.**
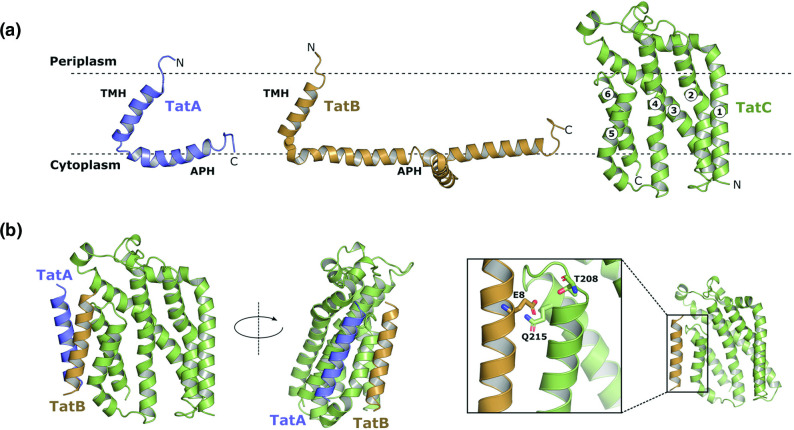
(**a**) Structures of TatA (blue), TatB (orange) and TatC (green). The transmembrane helices (TMHs) and amphipathic helices (APHs) of TatA and TatB are indicated, together with the TMH numbering in TatC. (**b**) A model for the resting state of the TatABC receptor complex, showing the interactions of the transmembrane helices, with the constituent subunits coloured as in (a). Polar cluster interactions between TatC residues T208 and Q215 (green) and TatB residue E8 (orange).

In prokaryotes, the Tat system has been best studied in the model bacterium *Escherichia coli *. The *E. coli* Tat translocase comprises three membrane proteins, TatA, TatB and TatC [10–13]. TatE, which is produced at very low levels, is a minor component that is functionally equivalent to TatA and is dispensable for Tat activity in laboratory conditions [10, 14]. Although TatA and TatB have differing functions during Tat transport, they derive from the same protein family [15]. They are monotopic membrane proteins with a very short N-terminal transmembrane helix, followed by an amphipathic helix located at the cytoplasmic side of the membrane, and an unstructured C-terminal tail [16, 17]. TatB proteins are generally bigger than TatA, having a longer amphipathic helical region and more extended tail [15] (Fig. 1a). TatC is the largest Tat component, consisting of six transmembrane domains with the N- and C-termini in the cytoplasm [9, 18] (Fig. 1a).

The Tat system is dynamic, and the active translocase assembles ‘on demand’, in a proton-motive force-dependent manner, triggered by functional interaction with substrate proteins [19–21]. In the resting state, the machinery comprises a TatABC ‘receptor complex’ with many further TatA molecules dispersed throughout the membrane [21–23]. The Tat receptor complex is multimeric and contains several copies of each Tat component, probably present in a 1 : 1 : 1 ratio [15, 22, 24]. Crosslinking, mutagenesis and co-evolution analysis has identified a critical binding site for TatA/TatB at transmembrane helix (TMH) 5 of TatC [15, 25, 26]. Binding to this site is mediated by a polar cluster of residues located at the C-terminal end of TatC TMH5 through to the start of TMH6 which co-ordinate a polar residue present, at equivalent positions, in the TMH of TatA or TatB. While either protein is able to occupy this binding site, in the resting state it is occupied by TatB [15] (Fig. 1b). Assembly of the translocase is initiated by interaction of the Tat receptor with a signal peptide. The conserved twin arginine motif is recognized by a negatively charged surface patch on TatC [9]. The signal peptide subsequently binds more deeply within the receptor, making extensive contacts with the TatB TMH [20, 25, 27–30]. This is accompanied by a rearrangement at the TMH5 binding site, where TatA replaces TatB, priming the recruitment of additional TatA protomers from the membrane pool [19–21, 31–34]. Substrate transport across the membrane is facilitated by the TatA oligomer, potentially through localized weakening of the cytoplasmic membrane [9, 16, 35].

Unexpectedly, a second binding site for TatA and TatB was also identified on TatC TMH6, through crosslinking analysis [26]. Again, each protein was able to bind to this site, but TatA was shown to occupy the site under resting conditions [26]. The functional relevance of this second binding site is currently unclear, because very few TatC-inactivating mutations have been identified that fall in this region of the protein. To explore the role of this binding site further we have undertaken extensive mutagenesis of TatC TMH6 and biochemical analysis of variant Tat receptor complexes. Our results indicate that this site is surprisingly robust to amino acid substitution and that even multiple bulky substitutions in positions that would be expected to disrupt binding interfaces do not abolish Tat activity.

## Methods

### Strains and plasmids

*E. coli* strain MC4100 [36] and the isogenic *tat* deletion mutants DADE (as MC4100, Δ*tatABCD*, Δ*tatE*) [37], DADE-P (as DADE, *pcn*B1 *zad*-981::Tn*10*d; Km^r^) [38] and MΔBC (as MC4100, Δ*tatBC*) [21] were used throughout this study. Strain XL1blue (Agilent) was used for all cloning steps. All plasmids used and constructed in this work are listed in Table 1.

**Table 1.** Plasmids used and constructed in this study For plasmids where substitutions were introduced by QuickChange, these are indicated in parentheses, including the names of specific primer pairs used to make them (listed in Table S1, available in the online version of this article), preceded by ‘QC’. Construction details for all other plasmids, which were made by other techniques and/or isolated from library screening, are included in the main text.

Plasmid	Description	Reference
pTAT1d	pUNI-PROM carrying *E. coli tatABC* with engineered restriction sites allowing facile replacement of each gene. ColE1 ori, Amp^r^	[55]
pJET1.2/blunt	pMB1ori, Amp^r^. Positive selection cloning vector	Thermofisher
pTTC1	pSU40 encoding TorAss-CAT fusion. Kan^r^	[55]
pESN5	As pTat1d, TatC-T216F (QC ESN25+ESN26)	This work
pESN6	As pTat1d, TatC-T216W (QC ESN25+ESN27)	This work
pESN7	As pTat1d, TatC-I220F (QC ESN15+ESN18)	This work
pESN8	As pTat1d, TatC-I220W (QC ESN16+ESN18)	This work
pESN11	As pTat1d, TatC-E227F (QC ESN19+ESN21)	This work
pESN12	As pTat1d, TatC-E227W (QC ESN19+ESN22)	This work
pESN13	As pTat1d, TatC-I220W, E227W (QC ESN18+ESN41)	This work
pESN15	As pTat1d, TatC-T216F, I220W, E227W (QC ESN43+ESN44)	This work
pESN16	As pTat1d, with *Esp*3I (QC ESN39+ESN40)	This work
pESN17	As pTat1d, with *Esp*3I and *Bsa*I sites removed	This work
pESN29	As pTat1d, TatC-C224R (QC ESN18+ESN65)	This work
pESN35	As pESN17, TatC-I220R	This work
pESN49	As pESN17, TatC-T216K (QC ESN25+ESN91)	This work
pESN65	As pESN17, TatC-L225Q (QC ESN18+ESN109)	This work
pESN69	As pESN17, *tatC*Δ(codons 201–246)::*Bsa*I(GCAT)-lacZα-*Bsa*I(AACG)	This work
pESN70	As pJET1.2/blunt, containing *Bsa*I(GCAT)-*tatC* (codons 201–246)-*Bsa*I(AACG)	This work
pESN82	As pESN17, *tatC* Δ(216-234)::*Bsa*I(GCAA)-lacZα-*Bsa*I(CTTT)	This work
pESN85	As pESN17, TatC-T216F, I220W, C224A, E227W (QC ESN44+ESN154)	This work
pESN97	As pESN17, TatC-P221R, C224A (QC ESN18+ESN160)	This work
pESN98	As pESN17, TatC-L217R, L218*, C224A (QC 161+ESN162)	This work
pESN99	As pESN17, TatC-L218F, C224A (QC ESN163+ESN164)	This work
pESN100	As pESN17, TatC-I220N, C224A (QC ESN18+ESN169)	This work
pESN101	As pESN17, TatC-M222L, C224A (QC ESN18+ESN170)	This work
pESN103	As pESN17, TatC-L225P	This work
pESN105	As pESN17, TatC-L217P, L218*, C224A (QC ESN167+ESN168)	This work
pESN107	As pESN17, TatC-C224A (QC ESN18+ESN189)	This work
pESN108	As pESN17, TatC-C224A, L225P (QC ESN18+ESN187)	This work
pESN109	As pESN17, TatC-C224A,E227G (QC ESN19+ESN191)	This work
pESN110	As pESN17, TatC-C224A,V230D (QC ESN19+ESN192)	This work
pAL7	As pESN17, TatC-A219R	This work
pAL8	As pESN17, TatC-F226R, G229D	This work
pAL9	As pESN17, TatC-F226R, F232E	This work
pAL11	As pESN17, TatC-A219K	This work
pAL12	As pESN17, TatC-L217K	This work
pAL13	As pESN17, TatC-Y223S (QC AL1+ESN18)	This work
pAL14	As pESN17, TatC-F226R (QC AL2+ESN19)	This work
pESN145	As pESN17, TatC-F226D, C224A (QC ESN19+ESN207)	This work
pESN147	As pESN17, TatC-F232E, C224A (QC ESN211+ESN212)	This work
pESN148	As pESN17, TatC-F232K, C224A (QC ESN209+ESN210 b)	This work
pAL15	As pESN17, TatC-M222R	This work
pAL16	As pESN17, TatC-L218E, M222R	This work
pAL17	As pESN17, TatC-T216E, M222R	This work
pAL18	As pESN17, TatC-T216L, M222R	This work
pAL19	As pESN17, TatC-T216C, M222R	This work
pAL20	As pESN17, TatC-F226D, F232K	This work
pAL22	As pESN17, TatC-T216K, L218F, M222P	This work
pESN152	As pESN17, TatC-P221R (QC ESN18+ESN199)	This work
pESN153	As pESN17, TatC-T216E (QC ESN25+ESN217)	This work
pESN154	As pESN17, TatC-T216L (QC ESN25+ESN218)	This work
pESN155	As pESN17, TatC-G229D (QC ESN19+ESN219)	This work
pESN168	As pESN17, TatC-I220R, C224A (QC ESN18+ESN93)	This work
pESN169	As pESN17, TatC-L218E (QC ESN220+ESN221)	This work
pESN175	As pESN17, TatC-M222P (QC ESN18+ESN225)	This work
pUC57Kan-TM6scan	As pUC57Kan, containing *Bsa*I(GCAA)-*tatC* (codons 216–235)-*Bsa*I(CTTT)	This work GenScript
p101CC4 AL9C, CF213C	pTAT101cysless, TatA-L9C, TatC-F213C	[26]
p101CC4 AL9C, CM205C	pTAT101cysless, TatB-L9C, TatC-F213C	[26]
p101CC4 BL9C, CM205C	pTAT101cysless, TatA-L9C, TatC-M205C	[26]
p101CC4 BL9C, CF213C	pTAT101cysless, TatB-L9C, TatC-M205C	[26]
pESN115	As pTAT101cysless, TatA-L9C, TatC-F213C, Δ(codons 209–246)::BsaI(CTGA)-*lacZ*α-BsaI(AACG)	This work
pESN116	As pTAT101cysless, TatB-L9C, TatC-F213C, Δ(codons 209–246)::BsaI(CTGA)-*lacZ*α-BsaI(AACG)	This work
pESN117	As pTAT101cysless, TatA-L9C, TatC-M205C, Δ(codons 209–246)::BsaI(CTGA)-*lacZ*α-BsaI(AACG)	This work
pESN118	As pTAT101cysless, TatB-L9C, TatC-M205C, Δ(codons 209–246)::BsaI(CTGA)-*lacZ*α-BsaI(AACG)	This work
pESN58	As pTAT101cysless, TatA-L9C, TatC-F213C, I220R (QC ESN93+ESN94)	This work
pESN59	As pTAT101cysless, TatA-L9C, TatC-M205C, I220R (QC ESN93+ESN95)	This work
pESN62	As pTAT101cysless, TatB-L9C, TatC-F213C, I220R (QC ESN93+ESN94)	This work
pESN66	As pTAT101cysless, TatB-L9C, TatC-FM205C, I220R (QC ESN93+ESN95)	This work
pESN126	As pTAT101cysless, TatA-L9C, TatC-F213C, T216F, I220W, E227W	This work
pESN127	As pTAT101cysless, TatB-L9C, TatC-F213C, T216F, I220W, E227W	This work
pESN128	As pTAT101cysless, TatA-L9C, TatC-M205C, T216F, I220W, E227W	This work
pESN129	As pTAT101cysless, TatB-L9C, TatC-FM205C, T216F, I220W, E227W	This work
pESN130	As pTAT101cysless, TatB-L9C, TatC-FM205C, P221R	This work
pESN131	As pTAT101cysless, TatA-L9C, TatC-F213C, P221R	This work
pESN132	As pTAT101cysless, TatB-L9C, TatC-F213C, P221R	This work
pESN133	As pTAT101cysless, TatA-L9C, TatC-M205C, P221R	This work
pESN134	As pTAT101cysless, TatB-L9C, TatC-FM205C, L225P	This work
pESN135	As pTAT101cysless, TatA-L9C, TatC-F213C, L225P	This work
pESN136	As pTAT101cysless, TatB-L9C, TatC-F213C, L225P	This work
pESN137	As pTAT101cysless, TatA-L9C, TatC-M205C, L225P	This work
pFAT75ΔA-*sufI*_FLAG_	pQE60 expression vector encoding *tatBC* and *sufI_his_* under control of the T5 promoter.	[8]
pFATBC_HIS_-*sufI*_FLAG_	As pFAT75ΔA-SufIFLAG, but with C-terminal His-tag on TatC	[8]
pESN324	As pFATBC-*tatC*_HIS_-*sufI*_FLAG_, TatC_HIS_-T216F, I220W, E227W	This work
pESN325	As pFATBC-*tatC*_HIS_-*sufI*_FLAG_, TatC_HIS_-P221R	This work
pESN326	As pFATBC-*tatC*_HIS_-*sufI*_FLAG_, TatC_HIS_-L225P	This work
pESN75	As pESN17, Δ*tatAB*::BsaI(CTAC)-*lacZ*α-BsaI(ACGA), TatC-I220R	This work
pESN76	As pJET1.2/blunt, containing BsaI(CTAC)-*tatAB*-BsaI(ACGA)	This work
pESN170	As pESN17, Δ*tatAB*::BsaI(CTAC)-*lacZ*α-BsaI(ACGA), TatC-A219K (QC ESN200+ESN201)	This work
pESN171	As pESN17, Δ*tatAB*::BsaI(CTAC)-*lacZ*α-BsaI(ACGA), TatC-A219R (QC ESN203+ESN204)	This work
pESN172	As pESN17, Δ*tatAB*::BsaI(CTAC)-*lacZ*α-BsaI(ACGA), TatC-M222R (QC ESN18+ESN213)	This work
pESN173	As pESN17, Δ*tatAB*::BsaI(CTAC)-*lacZ*α-BsaI(ACGA), TatC-P221R (QC ESN18+ESN199)	This work
pESN174	As pESN17, Δ*tatAB*::BsaI(CTAC)-*lacZ*α-BsaI(ACGA), TatC-L225P	This work
p101C*BCflag	Low copy vector for expression of *tatBC* producing TatB and C-terminally FLAG-tagged TatC	[42]
pESN181	As pC*BC101FLAG, TatC_FLAG_-Δ(codons 209–246)::BsaI(CTGA)-*lacZ*α-BsaI(AACG)	This work
pESN211	As pC*BC101FLAG, TatC_FLAG_-T216F, I220W, E227W	This work
pESN212	As pC*BC101FLAG, TatC_FLAG_-A219K	This work
pESN213	As pC*BC101FLAG, TatC_FLAG_-A219R	This work
pESN214	As pC*BC101FLAG, TatC_FLAG_-I220R	This work
pESN215	As pC*BC101FLAG, TatC_FLAG_-P221R	This work
pESN216	As pC*BC101FLAG, TatC_FLAG_-M222R	This work
pESN217	As pC*BC101FLAG, TatC_FLAG_-L225P	This work

*Indicates silent mutation.

### Site-directed mutagenesis

An overview of the mutagenesis strategies used in this work is given in Fig. S1. Site-directed mutagenesis of the *tatC* gene was performed with the modified QuickChange method described by Liu and Naismith [39]. All oligonucleotides used for mutagenesis, cloning and sequencing are listed in Table S1. The templates for whole-plasmid PCR amplification were either pTAT1d/pESN17 (WT *tatABC* template) or other constructs already bearing mutations in the case of multi-site mutants. Finished PCRs were treated with *Dpn*I prior to transformation. All constructs were confirmed by full sequencing of the *tat* genes before use.

### Construction of random mutagenesis libraries

Error-prone PCR (ePCR) was used to construct random mutagenesis libraries covering the region encoding TatC TMH6. We first constructed a template plasmid, pESN70 (Table 1), by cloning into pJET1.2/blunt (ThermoFisher) a PCR product amplified with primers ESN51+ESN52 (Table S1), encoding TatC TMH6 as well as adjacent parts of the TMH5–TMH6 periplasmic loop and the cytoplasmic C-terminus (codons 201–246). The primers also introduced *Bsa*I sites for Golden Gate assembly. Randomized PCR products were amplified from pESN70 using oligonucleotides ESN118 and ESN119 (Table S1). Reactions were carried out in 50 µl containing approximately 50 ng of template DNA alongside 0.2 mM each of dATP/dGTP, 1 mM each of dCTP/dTTP, 7 mM MgCl_2_, 0.4 mM each primer, 5 U GoTaq G2, 1× GoTaq buffer, and a range of MnCl_2_ concentrations (0, 0.1, 0.2, 0.3, 0.4, 0.5 or 0.6 mM) to modulate the mutation rate. The PCR cycle was: 94 °C, 2 min; 20× (94 °C, 30 s; 50 °C, 30 s; 72 °C, 30 s); 72, 1 min. PCR products were then treated with *Dpn*I for 1–2 h and then purified (QiaQuick; Qiagen) before use in library construction.

Golden Gate assembly [40] was used to clone each ePCR product into the recipient vector pESN69 (construction details below). Plasmid pESN69 is a modified version of pTAT1d in which the *Bsa*I and *Esp*3I sites have been removed by mutagenesis, and in which *tatC* codons 201–246 have been replaced with a *lacZ*α cassette flanked by *Bsa*I sites specifying quadruplets GCAT and AACG (codons 200 and 247 underlined) for seamless ligation. One-pot Golden Gate reactions were assembled in 20 µl as follows: approximately 250 ng pESN69, 80–100 ng ePCR, 1× T4 ligase buffer, 20 U *Bsa*I-HFv2 (NEB) and 20 U T4 ligase (NEB), and run in a thermal cycler with an initial 1 h 37 °C step followed by: 50× (37 °C, 2 min; 16 °C, 5 min); 50 °C, 5 min; 80 °C, 10 min.

Electrocompetent XL1blue cells (Agilent) were electroporated with 1 µl assembly mix and plated onto LB agar containing ampicillin along with 0.25 mM IPTG and 60 µg ml^−1^ X-Gal. We obtained between 80 000 and 130 000 colonies per transformation of which none were blue, indicating close to 100 % efficiency of assembly. Colonies were harvested in LB medium containing ampicillin, inoculated at an initial OD_600_ of 0.1 into fresh LB medium containing ampicillin and cultured for 5–6 h before harvesting 3–4×5 ml aliquots. Plasmid DNA was isolated from each aliquot, and the aliquots were pooled and used as the final library.

To determine the mutation rate in each library, 10–12 random colonies were picked and plasmid DNA isolated and sequenced (using primer ESN33, Table S1), from which we calculated a figure for the percentage error rate. As the codons targeted for mutagenesis covered approximately 140 nucleotides, we undertook functional screening using the library with a 0.6 % error rate (constructed from the ePCR amplified with 0.4 mM MnCl_2_) which yields on average 1 bp change in the target DNA.

ePCR was also used to construct random libraries of the *tatAB* bicistron to seek intergenic suppressors of all TatC TMH6 mutants. The construction of these libraries used the same design and procedures as described for *tatC*. Briefly, the *tatAB* template plasmid pESN76 was built by cloning a PCR product (ESN74+ESN75, Table S1) into pJET1.2/blunt. This plasmid served as template for ePCRs with MnCl_2_ concentrations of 0.1, 0.2, 0.3 and 0.4 mM, and the resulting PCR products were cloned into the Golden Gate vector, pESN75 (Table 1; see construction below) which was then moved into XL1blue for sequencing. We undertook functional selection with the library produced with 0.2 mM MnCl_2_, which returned an error rate of approximately 0.6 % equivalent to an average of 5 bp changes in the target DNA (primers ESN45 and ESN140 were used for sequencing). Suppressor libraries for all other inactive *tatC* TMH6 mutants were constructed in the isogenic vectors pESN170, pESN171, pESN172, pESN173 and pESN174 (see below). All transformations in XL1blue to build the libraries yielded approximately 1 000 000 transformants.

### Scanning mutagenesis library of the *tatC* TMH6 coding region

A synthetic scanning mutagenesis library of *tatC* codons 216–234 inclusive, comprising nominally 380 single mutants (stop codons were excluded), was synthesized by GenScript in vector pUC57Kan, with the synthetic fragments flanked by *Bsa*I sites. These fragments were moved via Golden Gate assembly (as described above, and using a 1 : 1 ratio of donor to recipient plasmids) into the recipient construct pESN82 (Table 1; construction details below), and electroporated into electrocompetent XL1blue cells.

### Library screening

The method for isolating inactive *tatC* mutants has been described elsewhere [41]. Briefly, mutagenesis libraries were introduced into strain DADE, pre-transformed with plasmid pTTC1, which encodes chloramphenicol acetyltransferase fused to a twin-arginine signal sequence. Transformants were first selected on LB containing ampicillin and chloramphenicol (the latter at 200–400 µg ml^−1^), and then replica-plated onto LB ampicillin and LB ampicillin plus 2% SDS to identify SDS-sensitive clones. Chl^R^ and SDS^S^ clones were patched on LB ampicillin and preliminarily screened by colony PCR (to quickly eliminate stop codon and frame-shift mutations). Clones harbouring mis-sense mutations were individually re-tested for SDS sensitivity and, once confirmed, used for plasmid preparation and re-transformation of *E. coli* XL1blue to segregate the pTAT1d-derivatives from pTTC1. These constructs were then fully sequenced, and again subjected to phenotypic testing after re-introduction into strain DADE.

Screening of ePCR *tatAB* libraries was performed as above except that libraries were moved into DADE cells and selected directly on 2 % SDS LB plates. While the synthetic scanning mutagenesis library of TatC TMH6 was designed to harbour only single codon substitutions, it also contained a small proportion of multiple mutants and codon deletions, due to synthesis errors. Inactive *tatC* mutants harbouring multiple substitutions mapping to TMH6 (codons 216–234) from either library were subsequently deconvoluted by engineering each mutation singularly into pESN17 by QuickChange (see above), followed by individual re-testing of these single mutants to determine whether the individual substitution resulted in TatC inactivation.

### Construction of recipient vectors for Golden Gate assembly

To construct Golden Gate recipient vectors for phenotypic characterization in DADE, pTAT1d was first modified by mutagenesis to remove the native *Bsa*I and *Esp*3I sites. Initially the *Esp*3I site was removed using Quickchange (ESN39+ESN40) to generate plasmid pESN16. Next, two PCR products were amplified using mutagenic oligonucleotides (Table S1) to suppress the two native *Bsa*I sites remaining in pESN16: oligonucleotides ESN47+ESN48 to remove the *Bsa*I site in *bla* and oligonucleotides ESN49+ESN50 to remove the *Bsa*I site just upstream of the *tat* promoter, respectively. Following digestion with *Dpn*I, these PCR products were Golden Gate-assembled using the engineered *Bsa*I sites present at their ends (Table S1), to generate plasmid pESN17.

The Golden Gate recipient vectors, pESN69 and pESN82, were derived from pESN17 in an equivalent manner using the NEBuilder HiFi DNA assembly kit (NEB). Plasmid pESN69 derived from the assembly of PCR products: ESN114+ESN115 from pESN17 (entire plasmid minus *tatC* codons 201–246) and ESN112+ESN113 on genomic DNA (gDNA) of *E. coli* MG1655 (*lacZ*α). pESN82 derived from PCR products: ESN152+ESN153 from pESN17 (entire plasmid minus *tatC* codons 216–234) and ESN128+ESN129 from pESN69 (*lacZ*α). Both assemblies were moved into XL1blue and screened on X-Gal-containing media for blue colour. The entire modified *tat* operon in both constructs was fully sequenced and correct cutting by *Bsa*I was confirmed by restriction digestion before further use.

The Golden Gate recipient vectors for the screening of *tatAB* ePCR libraries were made in two steps. First, pESN75 (C-I220R) and pESN174 (C-L225P) (Table 1) were constructed by NEBuilder HiFi assembly similar to that described for pESN69 above, i.e. by combining two PCR products, one for the backbone minus *tatAB* (ESN125+ESN126 on either pESN35 or pESN103, respectively) and one for *Bsa*I-flanked *lacZ*α (ESN123+ESN124 using gDNA of MG1655 as template). Vectors pESN170– pESN173, harbouring all other inactivating TMH6 mutations (Table 1), were then derived from pESN75 by QuickChange (Table S1). All constructs had their modified *tat* operon fully sequenced before use.

To simplify the making of plasmids for *in vivo* disulphide cross-linking (see below), we also constructed Golden Gate recipient vectors based on the previously described set of low-copy pTAT101cysless plasmids carrying unique pairwise combinations of Cys substitutions in either TatC TMH6 (F213C) or TMH5 (M205C) and either TatA (L9C) or TatB (L9C) [26]. Golden Gate recipient constructs, pESN115, pESN116, pESN117 and pESN118 (Table 1), were constructed by NEBuilder HiFi assembly from the following PCR products: ESN128+ESN129 on pESN69 (*lacZ*α) and either ESN139+ESN174 (F213C) or ESN139+ESN175 (M205C) on the appropriate pTAT101cysless templates (entire plasmid minus codons 209–246). The final constructs were screened and confirmed as described above for pESN69 and pESN82.

Finally, we also constructed a Golden Gate recipient vector, pESN181 (Table 1), to facilitate designing plasmids for close to native expression in MΔBC. pESN181 was constructed as described above for pESN115/116 using as the plasmid backbone construct p101C*BCflag [42], which encodes TatB alongside a C-terminally FLAG-tagged version of TatC. pESN181 was screened and confirmed as described for the other Golden Gate vectors.

### Construction of plasmids for co-purification

Plasmids pESN324, pESN325 and pESN326 are derivatives of pFATBC_HIS_-*sufI*_FLAG_ [42] carrying the TIE (triple T216F, I220W, E227W), P221R and L225P mutations in *tatC*, respectively. These were obtained using the NEBuilder HiFi DNA assembly kit to join the following PCR products: ESN114+ESN180 from pFATBC_HIS_-*sufI*_FLAG_ (entire plasmid minus *tatC* TMH6 coding sequence) and ESN178+ESN182 from either pESN15, pESN152 or pESN103 (Tables 1 and S1). Inserts were sequenced with forward primer ESN83 to confirm the presence of the correct TMH6 mutation as well as that of the histidine tag codons and of the start of *sufI*. As part of the screening process, a plasmid was isolated that carried a single nucleotide deletion at the 5′ junction between the two PCR products, causing a frameshift upstream of the TMH6 coding region of *tatC*. This construct, simply named ‘Δ*tatC*’, was retained as a further negative control for the co-purification assays.

### Construction of plasmids for *in vivo* disulphide cross-linking

All constructs for *in vivo* disulphide cross-linking (Table 1) were made in two steps. First, QuickChange was used to introduce the C224A substitution in pTAT1d-based constructs: ESN44+ESN154 on pESN15 to give pESN85 (TIE), ESN18+ESN93 on pESN17 to give pESN168 (I220R), ESN18+ESN160 on pESN17 to give pESN97 (P221R), and ESN18+ESN187 on pESN17 to give pESN108 (L225P) (Tables 1 and S1). In the second step, these plasmids were used as templates for two sets of PCRs that were Golden Gate-assembled into the final pTAT101cysless-based recipients (see above): ESN132+ESN177 for pESN115 and pESN116 (F213C), and ESN132+ESN176 for pESN117 and pESN118 (M205C). Final constructs (Table 1) were sequenced with primer ESN104 to confirm correct assembly and the presence of the expected TMH6 mutation and Cys replacements.

### Construction of plasmids for expression in strain MΔBC

ESN132+ESN176 PCR products amplified from plasmids pESN15, pESN35, pESN103, pESN152, pAL7, pAL11 and pAL15 were Golden Gate-assembled into pESN181 (see above). Final constructs (Table 1) were confirmed as described above for the cross-linking plasmids.

### Preparation of membrane fractions

Membrane fractions of *E. coli* cultures were prepared as described previously [43]. Briefly, overnight cultures grown from single colonies of fresh transformants were refreshed in LB supplemented with the appropriate antibiotics (if required) to an initial OD_600_ of 0.05 and grown at 37 °C with shaking until an OD_600_ of 0.5–1. Cells were then harvested from a volume of 25–50 ml (depending on the plasmid copy number), resuspended in 1 ml of the buffer required for each particular application (see below) and supplemented with protease inhibitors (Roche), and then disrupted by sonication. After clarification, membrane fractions were separated from the cell lysate by ultracentrifugation and finally resuspended in 30–60 µl Buffer 2 (50 mM Tris-HCl, pH 7.5, 5 mM MgCl_2_, 10 % glycerol) unless indicated otherwise.

### Blue-Native PAGE

Blue-Native (BN) PAGE was performed as described previously [42] except that digitonin-mediated solubilization was performed overnight. Briefly, membrane fractions were prepared as described above, but with PBS used for cell resuspension and sonication, and Buffer A (50 mM NaCl, 50 mM imidazole, 2 mM 6-aminohexanoic acid, 1 mM EDTA; pH 7.0) used for final resuspension. After solubilization of the membrane fractions with 2 % digitonin, the solubilized material was recovered by ultracentrifugation and mixed with 5 % glycerol and 0.25 % Coomassie blue G-250 buffer. Approximately 20 µg of total protein was loaded per lane. Gels were subsequently analysed by Western blotting using an anti-TatC antibody [44]. Blots were developed with ECL (BioRad).

### Co-purification of TatC_HIS_, TatB and SufI_FLAG_

For co-purification of TatC_HIS_, TatB and SufI_FLAG_, cultures of DADE-P carrying either pFAT75ΔA-*sufI*_FLAG_ (control plasmid) or pBC_HIS_-*sufI*_FLAG_ derivatives were grown as for membrane fraction preparation, but in the presence of 1 mM IPTG. After sonication, clarified cell lysates were solubilized overnight with 1.5 % digitonin in Buffer 1 (20 mM Tris-HCl, pH7.5, 200 mM NaCl, 50 mM imidazole, 10 % glycerol) and the soluble fraction separated by ultracentrifugation. TatC_HIS_ complexes with TatB and SufI_FLAG_ were captured with Ni-NTA magnetic beads (ThermoFisher), which were washed three times with wash buffer (20 mM Tris-HCl, pH 7.5, 200 mM NaCl, 25 mM imidazole, 0.5 % digitonin) before elution with 1× SDS-PAGE loading buffer (Brand) by mild thermal treatment (53 °C for 10 min). Samples were then run on SDS-PAGE gradient gels (BioRad) and analysed by Western blotting with anti-TatB [15], anti-His (Invitrogen) or anti-FLAG (Sigma) antibodies. Blots were developed with ECL (BioRad).

### *In vivo* disulphide cross-linking

*In vivo* disulphide cross-linking was performed as described [26]. Briefly, strain DADE harbouring pTAT101cysless-based plasmids (Table 1) were prepared for membrane fractions as outlined above. Once at an OD_600_ of 0.5, each 50 ml culture was split in two equal volumes which were reacted with either copper phenanthroline (CuP) 1.8 mM or 10 mM DTT for 1 min before being washed with ResB buffer (20 mM Tris-HCl, 200 mM NaCl, pH 7.5) and finally resuspended in 1 ml ResB supplemented with 12 mM EDTA and 8 mM *N*-ethylmaleimide (NEM) to stop the reaction. Samples were then subjected to membrane fractionation as outlined above. Ten microlitres of each sample was used for SDS-PAGE and Western blotting.

### TatC expression levels in strain MΔBC

Membrane fractions were prepared as described above from strain MΔBC carrying pC*BC101FLAG derivatives (Table 1) using ResB (see above) as wash buffer. Ten microlitres per sample was used for SDS-PAGE and Western blotting. Monoclonal anti-FLAG antibody M2 (Sigma) was used for detection of TatC_FLAG_.

### Molecular modelling and simulations

Molecular modelling of the TatABC multimer was carried out as described previously [15]. Multimers were built using TatA–TatC/TatB–TatC disulphide cross-links as unambiguous constraints for docking using HADDOCK [45]. For all models, TatA was modelled from residues G2 to G21, TatB from residues F2 to G21 and TatC from residues T11 to F235. The CHARMM-GUI membrane builder functionality [46] was used to set up the systems for molecular dynamics (MD) simulations. The TatABC complexes (WT and P221R, L225 and TIE mutants) were inserted in a bilayer containing 25 % 1-palmitoyl, 2-oleoyl phosphatidylglycerol and 75 % 1-palmitoyl, 2-oleoyl phosphatidylethanolamine and solvated with TIP3P waters and 0.15 M NaCl. All MD simulations were performed using GROMACS 2021 [47] and CHARMM36m force field [48] with a timestep of 2 fs. The input files for minimization and equilibration provided by CHARMM-GUI were used. The system was first minimized followed by a series of equilibration steps consisting of gradual removal of the restraints from lipid and protein atoms for a total time of 2 ns. Three repeats of 100 ns of unrestrained atomistic MD simulations, for each configuration of the molecular system were performed. All simulations were executed at 300 K and 1 bar with protein, lipids and solvent separately coupled to an external bath, using the velocity-rescale thermostat [49] and Parrinello-Rahman [50] barostat. All bonds were constrained with the LINCS algorithm [51]. The long-range electrostatic interactions were computed with the Particle Mesh Ewald method [52], while a Verlet cut-off method was used to compute the non-bonded interactions. All images were generated using PyMOL [53].

## Results

### Bulky substitutions at the TatA/TatB binding site on TatC TMH6 do not abolish Tat function

**Fig. 2.**
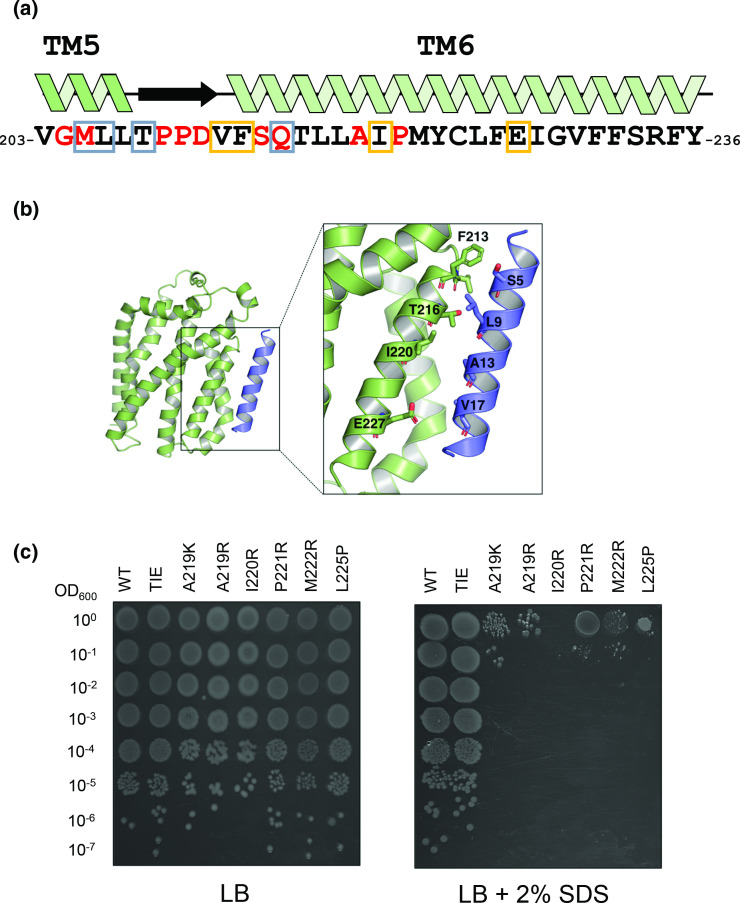
Mutagenesis of TatC TMH6. (**a**) Amino acid sequence of TatC TMH6 and flanking regions. Inactivating substitutions falling in this region of TatC that have been identified previously [15, 41, 57] are indicated by red font. Residues boxed in grey have been shown by cysteine-substitution and crosslinking (M205, L206 [26]) or co-evolution analysis, molecular simulations and mutagenesis [15] to form the TatA/TatB TMH5 binding site. Residues shown boxed in yellow are part of the TMH6 binding site [26]. (**b**) Structural model of TatA bound at the TMH6 binding site. (**c**) Phenotypic characterization of TatC single amino acid TMH6 variants. Spot tests of strain DADE (Δ*tatABCD*, Δ*tatE*) carrying pTAT1d derivatives producing TatA, TatB and the indicated amino acid TMH6 variant in TatC. Strains were re-grown from overnight cultures in liquid medium to an OD_600_ of 1, decimally diluted and spotted (10 μl) on LB medium with or without added 2 % SDS, and finally incubated overnight aerobically at 37 °C.

Structural analysis of *Aquifex aeolicus* TatC identified that TMH5 and TMH6 contain fewer amino acids than conventional TM helices, and therefore are slightly shorter in length than normal TM helices. This region of TatC proteins comprises a number of highly co-evolving residue pairs, which suggests tight structural interplay between both TMHs [9]. A primary binding site for TatA/TatB has been characterized along TMH5 of TatC, with residues in the adjacent loop and the top of TMH6 also forming a key part of the site; this is referred to as the polar cluster site (Fig. 2a). The TMH6 binding site is largely formed by residues in TMH6, including F213, I220 and E227 (Fig. 2a, b, all numbering is for *E. coli* TatC). Initially we took a targeted approach in an attempt to disrupt the TatA/TatB binding site on TatC TMH6. We selected residues that fall on the face of TatC TMH6, which have been shown to interact with TatA by crosslinking studies [26] (Fig. 2b). We also mutated each of T216, I220 and E227 singly and in combination to bulky phenylalanine (T216F) or tryptophan (I220W, E227W). We then tested for Tat transport activity by plating cells producing these variants of TatC onto LB containing 2 % SDS. Complete block of the Tat pathway results in the failure to export the two cell wall amidases, AmiA and AmiC, to the periplasm, and the resultant defect in cell wall remodelling makes cells exquisitely sensitive to killing by detergent [54]. However, as seen in Fig. 1(c), even a TatC variant where T216, I220 and E227 are triply-substituted (‘TIE’ mutation) did not abolish Tat activity and was able to support robust growth on SDS.

### Random mutagenesis of the TatC TMH6 coding region

Next, we turned to random mutagenesis as an unbiased approach to isolate inactivating amino acid substitutions in TatC TMH6. To date, only two inactivating substitutions, A219E and L225P, have been reported [41]. To identify further inactivating substitutions, we initially used error-prone PCR to construct a random library of 90 000 clones containing mutations covering the coding region for TatC TMH6 and flanking regions, as described in the Methods. To isolate amino acid exchanges that compromise the activity of TatC, we used a two-step approach that we have previously successfully employed to identify inactivating substitutions in Tat components [41]. The TorA-CAT reporter protein comprises the twin-arginine signal sequence of the Tat substrate protein TorA, fused to chloramphenicol acetyltransferase [55]. Export of this fusion protein to the periplasm by the Tat pathway renders *tat* ^+^ cells sensitive to growth inhibition by chloramphenicol. This is because detoxification of chloramphenicol by CAT requires acetyl coenzyme A as a co-substrate, which is only found in the cytoplasm. Substitutions that reduce or abolish Tat transport allow cytoplasmic CAT to accumulate, conferring resistance to chloramphenicol. As this initial screen does not differentiate substitutions that reduce Tat activity from those that fully abolish it, all chloramphenicol-resistant colonies from the first round of screening were subsequently plated onto LB containing 2 % SDS to identify substitutions that completely abolish TatC activity.

Following extensive screening of the library, we found 101 mutant clones that inactivated the function of TatC. Of those, 53 contained a premature stop codon and 10 had frameshifts (Table S2). The amino acid substitutions that were present in the remaining 38 clones are listed in Table 2. A number of substitutions were isolated that fell in the C-terminal region of TMH5 and the TMH5–TMH6 loop region, including M205K/R and Q215R, that have been isolated previously [41]. We also isolated seven clones harbouring the TatC L225P substitution, including three where it was the sole mutation present. For the clones with multiple substitutions, we followed up by making the single mutations L217P, L217R, I220N, P221R, M222L, Y223S, C224R, L225Q, E227G and V230D. From this deconvolution, P221R was the only further single substitution that inactivated TatC.

**Table 2.** Inactivating amino acid substitutions in TatC isolated from an error-prone PCR library covering TMH6 A dash indicates that the clones conferred full sensitivity to SDS (as determined by the inability of a 10 µl of culture of an OD_600_ of 1 to grow on solid medium containing 2 % SDS). A ‘+/-’ sign indicates that clones conferred partial sensitivity to SDS (as determined by an inability to support growth beyond a 10^−4^ dilution of an OD_600_=1 culture). Substitutions shown in bold have been isolated previously and shown to inactivate the function of TatC [41]. Those underlined indicate that changes at these amino acid positions are known to inactivate TatC when substituted to something other than that identified here.

TatC substitution	Growth on SDS	TatC substitution	Growth on SDS
**M205K,** L225Q	**+/−**	**Q215R** (isolated five times independently)	**–**
M205T, L207P, D211V	**+/−**	**Q215R**, Y236H	**–**
Q215K, I220V	**+/−**	V212I, **Q215R**	**–**
G204W, L217P	**+/−**	M205L, **Q215R**	**–**
L217R, F232S	**+/−**	**Q215R**, K239N	**–**
I220N, M222L, E227G, V230D	**+/−**	V212A, **Q215R**, Y223H, V230D	**–**
L217P, M222L, E227V	**+/−**	D211E, **Q215R**, T216K, L217Q	**–**
D211V, C224R, K239E	**+/−**	D211G, **Q215R**	**–**
P210T, Y223S, C224R	**+/−**	M205I, **Q215R**	**–**
V203I, L207Q, T208A, F213Y, C224R	**+/−**	**Q215R**, F235S	**–**
M205V, L206S, P221R	**–**	**Q215R**, C224G, E227D, F235L	**–**
V203A, G204R	**–**	**L225P** (isolated three times independently)	**–**
G204R, I228V	**–**	M222V, **L225P**, E227G	**–**
V203D, G204R, V212A, L218K, F226L	**–**	**L225P**, E227G, K239I, E244G	**–**
**M205R**	**–**	M222T, **L225P**, F235S, G240W	**–**
**M205R**, G229S	**–**	F213Y, **L225P**	**–**

### A scanning mutagenesis library permits identification of further inactivating substitutions in TatC TMH6

While ePCR is a useful approach to generate point mutations, not all substitutions are likely to be covered because some amino acid exchanges require two base changes at a single codon. Therefore, to ensure we screened all possible amino acid substitutions at each position, we had a commercial library synthesized that encoded every single amino acid change at TatC residues 216–234, excluding stop codons. We opted to start the mutagenesis from residue 216 since some amino acids prior to this position also play key roles in the TMH5 binding site. Screening this library identified further substitutions that inactivate TatC (Table 3), including positively charged substitutions at A219 and arginine substitutions at I220 and M222. Fifteen frame shifts and a small number of multiple substitutions were also isolated that represent synthesis errors and are present at very low levels in the library but are amplified in our screen because they inactivate TatC. From the small number of multiple substitutions, we constructed individual TatC T216L, T216K, T216E, L218E, L218F, M222P, F226R, F226D, G229D, F232K and F232E substitutions, all of which retained activity. A T216C substitution has been constructed previously and also shown to be functional [26].

**Table 3.** Inactivating amino acid substitutions in TatC isolated from a scanning mutagenesis library of TMH6 A dash indicates that the clones conferred full sensitivity to SDS (as determined by the inability of a 10 µl of culture of an OD_600_ of 1 to grow on solid medium containing 2 % SDS). A ‘+/-’ sign indicates that clones conferred partial sensitivity to SDS (as determined by an inability to support growth beyond a 10^−4^ dilution of an OD_600_ of 1 culture) (typically, sensitivity showed between dilutions of −2 and −4).

TatC substitution	Growth on SDS	TatC substitution	Growth on SDS
L217K (isolated two twice)	**+/−**	T216C, M222R	**–**
I220R (isolated six times)	**–**	L218E, M222R	**–**
A219R (isolated six times)	**–**	F226D, F232K	**–**
A219K (isolated twice)	**–**	F226R, G229D	**–**
M222R	**–**	F226R, F232E	**–**
T216E, M222R (isolated twice)	**–**	T216K, L218F, M222P	**–**
T216L, M222R	**–**		

Together our mutant screens identified A219K, A219R, I220R, P221R, M222R and L225P as single substitutions inactivating TatC, and Fig. 1(c) shows the SDS growth phenotype associated with each of these substitutions.

Previously we have used suppression genetics to isolate mutants in *tatB* that restore activity to inactivating substitutions in TatC, providing mechanistic insight into their functions [41, 42]. We therefore designed mutant libraries of *tatAB* by ePCR and used these in attempts to suppress each of our inactive TatC substitutions, selecting for growth on LB containing SDS. However, despite numerous rounds of screening we were unable to isolate any robust suppressors for any of these substitutions. Moreover, the strong TatB suppressor, TatBF13Y, which restores Tat activity to substitutions in either TatC signal peptide binding site, or to inactive Tat signal peptides [42], was unable to restore detectable activity to any of our TatC mutants.

### Positively charged amino acids at residues 219 and 220 destabilize TatC

**Fig. 3.**
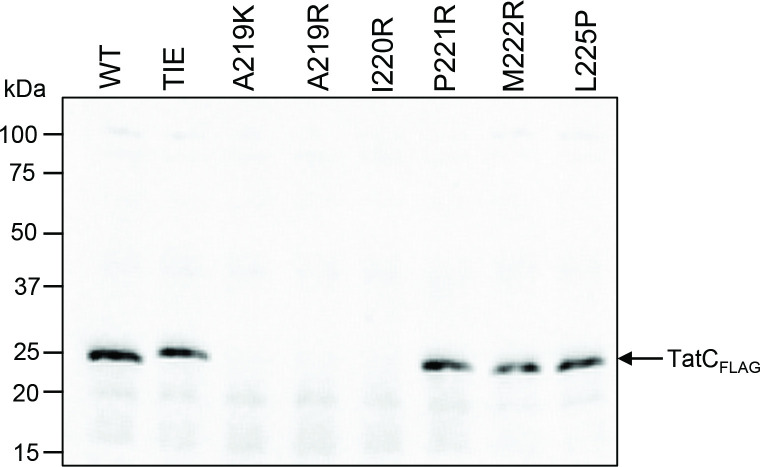
Membrane accumulation levels of TatC single amino acid TMH6 variants expressed from a low-copy vector. Western blots of membrane fractions from MΔBC (as MC4100, Δ*tatBC*) cultures harbouring derivatives of the low-copy construct pC*BC101FLAG expressing TatB and the indicated amino acid TMH6 variant in C-terminally FLAG-tagged TatC. Crude membrane fractions were prepared from exponentially growing cultures as described in the Methods, blotted and probed with an anti-FLAG monoclonal antibody. An equal amount of total protein was loaded in each lane.

**Fig. 4.**
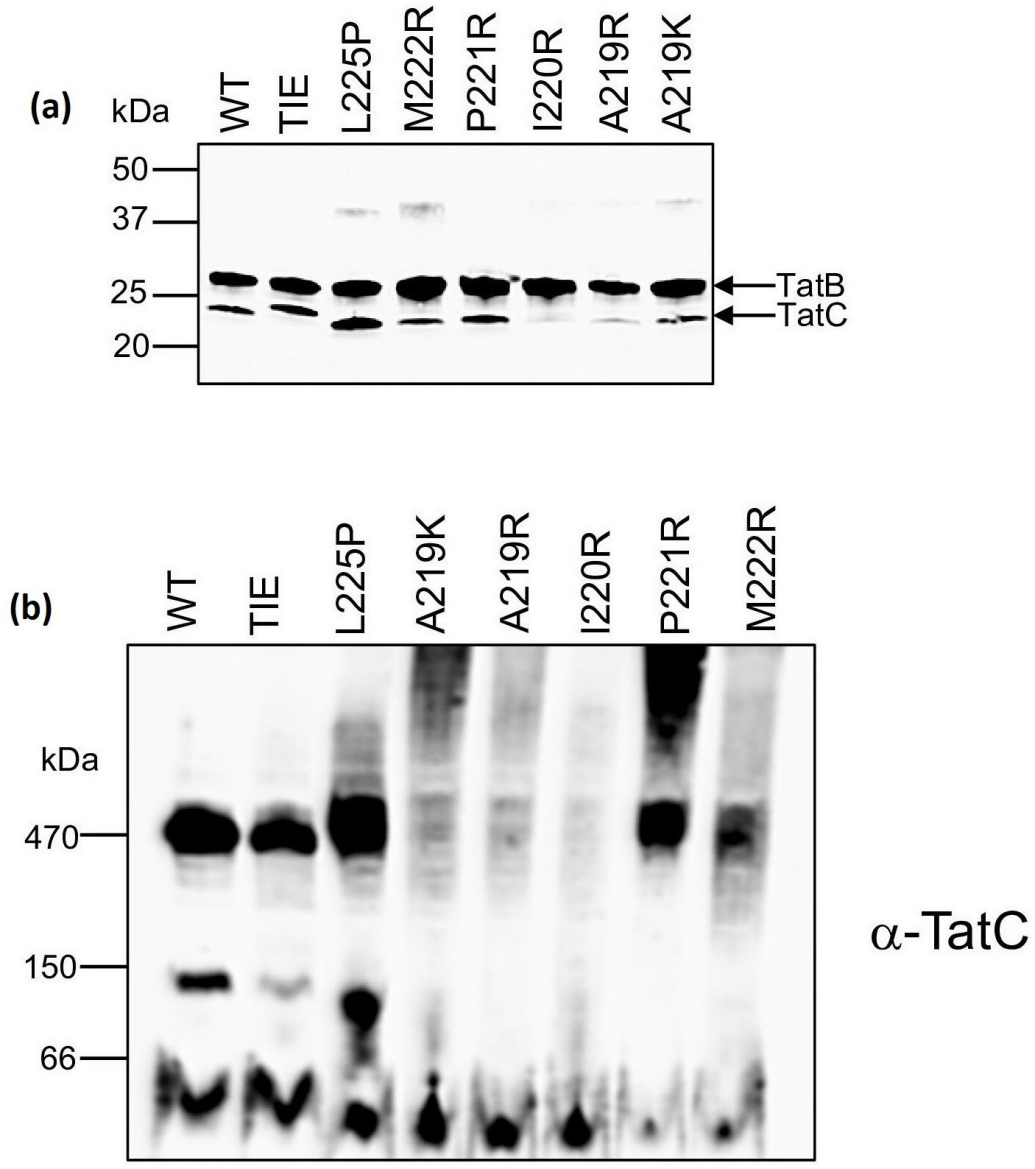
BN-PAGE analysis of Tat complexes containing TatC TMH6 variants. (**a**) Crude membranes from strain DADE (Δ*tatABCD*, Δ*tatE*) producing the indicated TatC variants alongside wild-type TatA and TatB from plasmid pTAT1d were solubilized by addition of 2 % (w/v) digitonin and analysed by (**a**) SDS-PAGE and Western blotting with a mix of anti-TatB and anti-TatC antibodies to assess protein levels; and (**b**) BN-PAGE (4–16% Bis-Tris Native PAGE gels; 20 µg solubilized membrane per lane) followed by Western blotting with an anti-TatC antibody as indicated.

We next biochemically characterized the Tat system harbouring the inactivating substitutions. We first asked whether any of the mutations in TMH6 destabilized TatC. Fig. 3 indicates that the A219K, A219R and I220R variants of FLAG-tagged TatC are indeed unstable and cannot be detected in membrane fractions, probably explaining their lack of activity. When we expressed these substitutions from a higher copy number vector, we again observed that these alleles are destabilized relative to wild-type TatC (Fig. 4a), although we were able to faintly detect some TatC from these constructs.

The Tat receptor complex has been extensively characterized by BN gel electrophoresis, and shown to migrate just above 440 kDa on 4–16 % gradient gels (e.g. [56]). We therefore tested whether any of the TMH6 substitutions affected migration of the complex. Fig. 4(b) shows that a TatC-reactive complex with a size of around 470 kDa was detected when TatC was wild-type, and also when it harboured the TIE triple substitution (which does not abolish Tat function) or any of the P221R, M222R or L225P inactivating substitutions. As expected, given the lack of stability of TatC A219K, A219R and I220R, no TatC-containing complexes could be detected following BN-PAGE. We conclude that the P221R, M222R and L225P substitutions of TatC do not affect assembly of the Tat receptor.

### TatBC complexes containing the P221R and L225P TatC substitutions can still interact with a Tat substrate

**Fig. 5.**
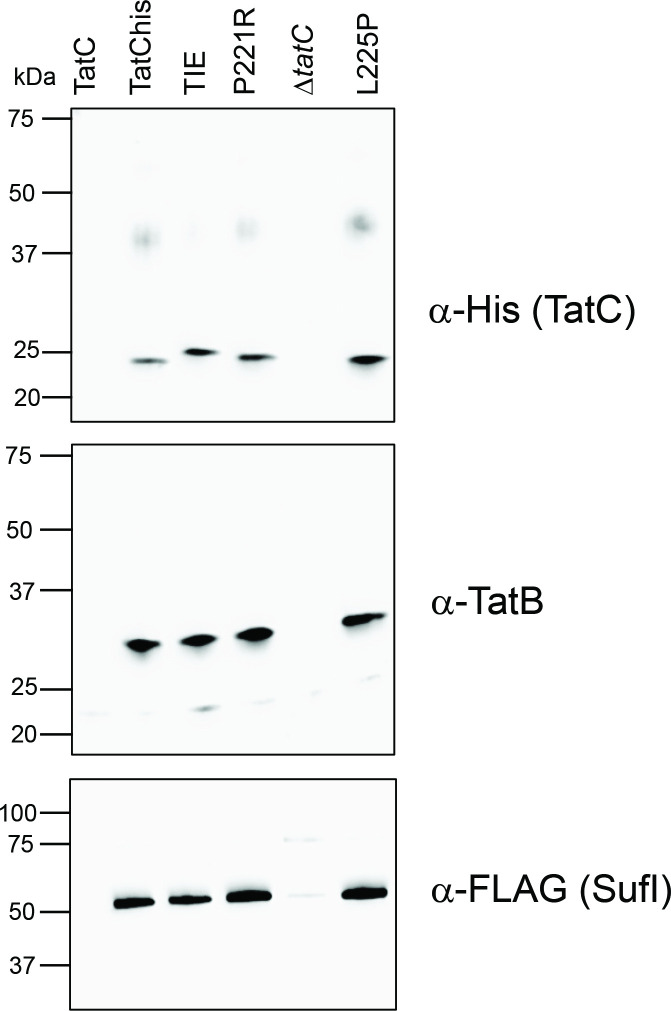
Co-purification of TatB and variant TatC with the Tat substrate SufI. Strain DADE-P (Δ*tatABCD*, Δ*tatE*, *pcnB1*) producing the indicated TatC_HIS_ variants alongside wild-type TatB and the Tat substrate SufI_FLAG_ from plasmid pFATBC_HIS_-*sufI*_FLAG_ were inoculated from overnight cultures at a starting OD_600_ of 0.05 and grown for 3 h in the presence of 1 mM IPTG after which membrane fractions were produced as described in the Methods. Membranes were solubilized by addition of 2 % (w/v) digitonin and incubated with Ni-NTA-magnetic beads to separate TatC_HIS_-containing complexes. Affinity-bound complexes were eluted by the thermal treatment of the beads and analysed on SDS-PAGE with anti-His (for TatC), anti-TatB and anti-FLAG antibodies. ‘*tatC*’: DADE-P harbouring pFATΔA-*sufI*_FLAG_ expressing WT TatC without a His-tag [8]; ‘*ΔtatC*’: DADE-P carrying a pFATBC_HIS_-*sufI*_FLAG_ derivative with a PCR-induced frameshift within the TMH6 coding region of *tatC*_HIS_, used here as a further negative control.

A key function of the Tat receptor complex is the interaction with substrate proteins, mediated through binding with their twin-arginine signal peptides. We therefore sought to determine whether the P221R and L225P TatC substitutions were inactive because they prevented interaction with the Tat substrate, SufI. We co-produced TatB and C-terminally His-tagged TatC alongside SufI-FLAG, isolated membrane fractions, solubilized with detergent and pulled out TatC–His complexes. Fig. 5 shows that FLAG-tagged SufI specifically co-purified with TatB and His-tagged TatC, even when the P221R or L225P substitutions were present, and therefore these substitutions do not impede SufI interaction.

### Disulphide crosslinking reveals that inactivating TMH6 substitutions do not prevent binding of TatA or TatB to the TMH5 or TMH6 sites

**Fig. 6.**
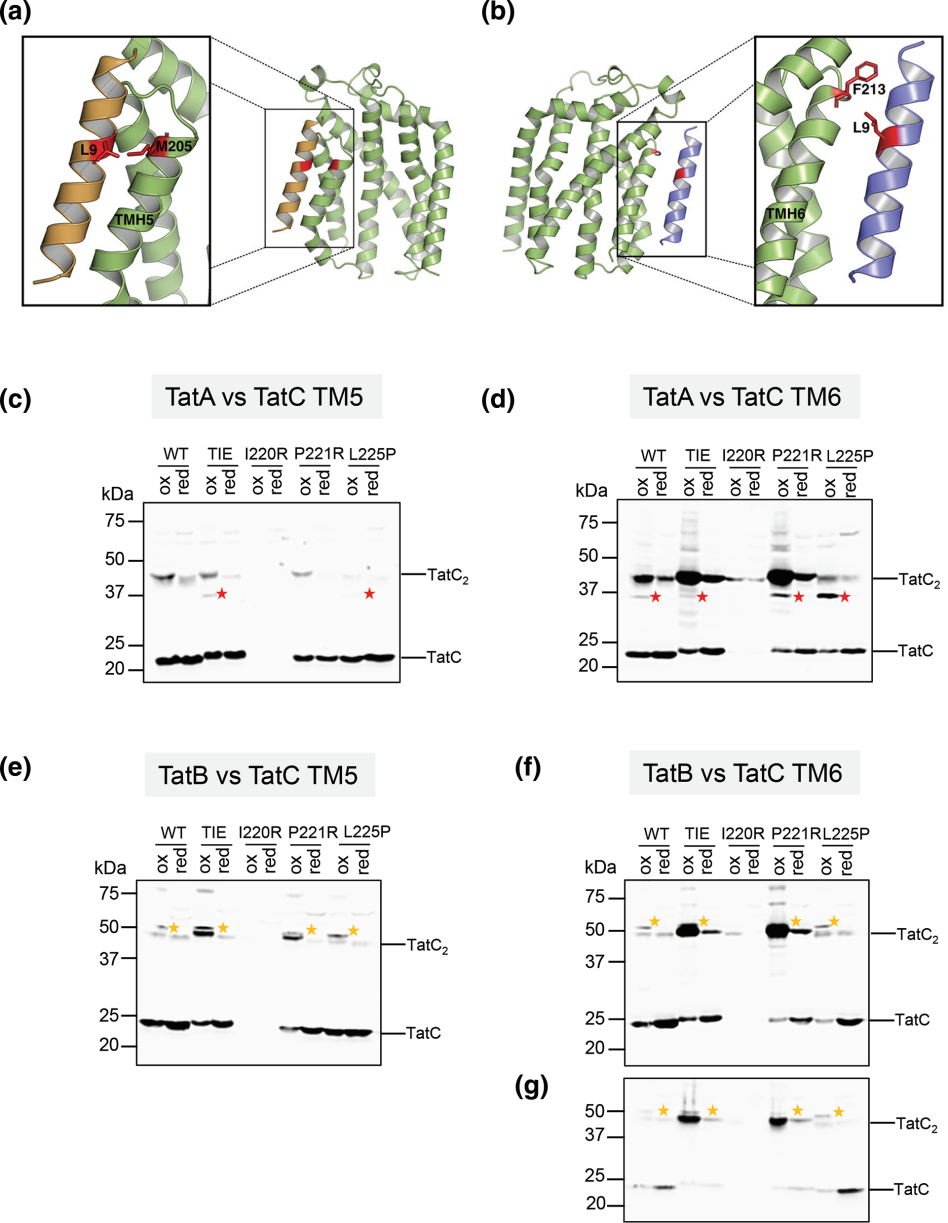
*In vivo* disulphide cross-linking between TatC harbouring TMH6 substitutions and TatA or TatB. (**a and b**) Diagnostic crosslinks between TatA/TatB and TatC can be used to probe occupancy of (**a**) the TMH5 site (L9C substituted TatA or TatB and M205C TatC) and (**b**) the TMH6 site (L9C substituted TatA or TatB and F213C TatC). (**c–g**) Membranes from strain DADE (Δ*tatABCD*, Δ*tatE*) producing the indicated TatC variants and either the M205C or F213C substitutions alongside L9C variants of either TatA or TatB from the low-copy plasmid pTAT101cysless were analysed by anti-TatC Western blotting after exposure of whole cells to either 1.8 mM CuP (oxidizing, ‘ox’) or 10 mM DTT (reducing, ‘red’). Crosslinks are shown between (**c**) TatA[L9C] and TatC[M205C]; (**d**) TatA[L9C] and TatC[M213C]; (**e**) TatB[L9C] and TatC[M205C]; (**f**) TatB[L9C] and TatC[F213C]. (**g**) Is a reload of the membranes from (**f**) with reduced exposure to prevent signal saturation. In each case red asterisks mark the positions of TatA–TatC crosslinks and yellow asterisks the TatB–TatC crosslinks.

Finally, we tested whether any of the inactivating I220R, P221R or L225P substitutions, or the functional ‘TIE’ triple substitution of TatC blocked the ability of TatA or TatB to occupy either of the two binding sites. The occupancy of these sites can be probed using site-specific crosslinking; the presence of TatA or TatB in the TMH5 site is shown by a disulphide crosslink between an L9C substitution in TatA or TatB and an M205C substitution in TatC (Fig. 6a). Conversely, the presence of TatA/TatB in the TMH6 site is detected through a crosslink between the same L9C substitutions and an F213C substitution in TatC (Fig. 6b).

Comparing Fig. 6(c) and (d) indicates that when TatC is otherwise wild-type, TatA is largely detected in the TMH6 binding site, as reported previously [26]. As expected, the I220R substitution resulted in TatC instability and barely any protein could be detected. However, for both the P221R and L225P substitutions, TatA is present at the TMH6 site, indicating that this binding site has not been grossly disrupted by the substitutions. Conversely, very little TatA is detected in the TMH6 site when the triple bulky amino acid substitutions (‘TIE’) are present. However, this binding site cannot be completely disrupted by these mutations because we were able to detect some TatB bound there (Fig. 6f, g). Likewise, neither the P221R nor L225P substitutions completely abolished the ability of TatB to interact with the TMH5 or TMH6 sites. Finally, we note that while there is some TatA at the TMH5 site for the L225P substitution, we could not detect TatA in this site when the TatC P221R substitution was present.

### MD simulations of TatABC complexes

**Fig. 7.**
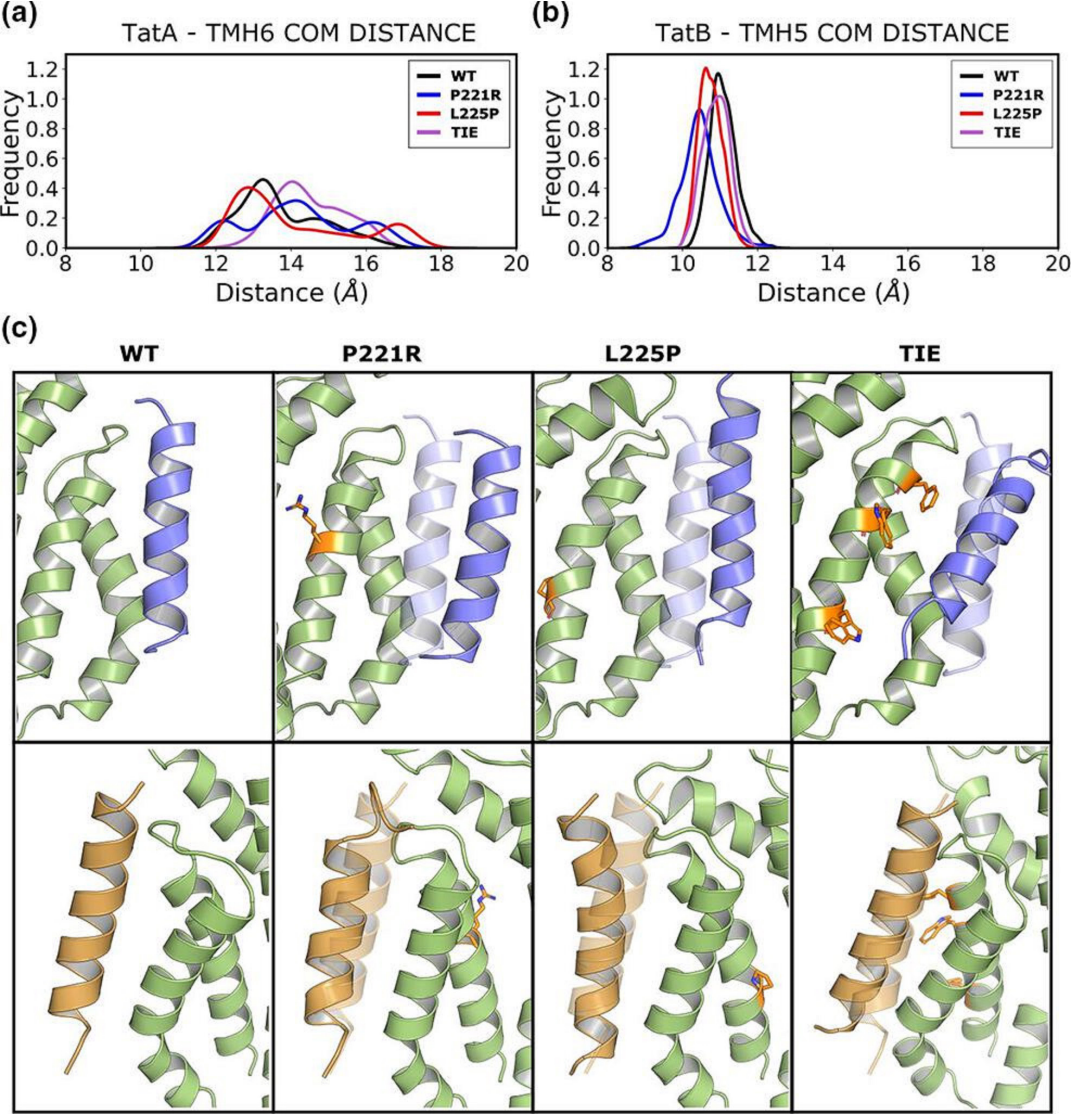
Molecular simulations of the interactions of TatA with TatC TMH6 for the WT and P221R, L225P and TIE mutants. (**a**) Distances between TatA and TatC TMH6. (**b**) Distances between TatB and TatC TMH5. The centre of mass of the transmembrane helices was used to calculate the distances. (**c**) Snapshots of the MD simulations showing the displacement of TatA (blue) and TatB (orange) from the TatC (green) TMH6 and TMH5 binding sites, respectively. The three TatC mutants are shown in comparison with the WT.

To structurally test the impact of the mutations on modelled complexes of *E. coli* TatABC, we ran three repeats of MD simulations of wild-type, P221R, L225P and TIE variants of TatC, with TatA in the TMH6 binding site and TatB in the TMH5 binding site. For the wild-type simulations, both single-pass TMHs remained bound to their respective sites over the course of the simulations, with some deviation observed for TatA bound to TMH6. However, at the TMH6 site, all mutant simulations showed a greater separation between TatA and TatC, with the bulky-substituted TIE variant showing the greatest separation (Fig. 7a), in agreement with the very weak TatA L9C–TatC F213C crosslinking we observed when these mutations were present (Fig. 6d). Snapshots of the MD simulations, shown in Fig. 7(c), indicate that for all three of the TatC variants there is TatA disassociation from the TMH6 site, whereas in contrast, TatB remains bound to the TMH5 site for both wild-type and mutant simulations.

## Discussion

In this study we have probed the role of TatC TMH6 for function of the Tat pathway. Previous work has shown that the N-terminal end of TMH6 contributes to a key binding site for TatA/TatB, and that this site (here termed the TMH5 site) is mechanistically essential for Tat activity [15, 26, 41]. The opposing face of the helix forms a separate interaction interface for TatA/TatB, the TMH6 site, the relevance of which is unclear [26]. In an effort to determine whether this binding site is critical for Tat transport, we undertook extensive mutagenesis of TMH6 to identify substitutions that blocked activity. From this we identified six single substitutions that rendered TatC inactive, of which only three produced stable TatC protein. These three TatC variants, P221R, M222R and L225P, did not affect assembly of the Tat receptor complex, and we confirmed that two of those, P221R and L225P, also did not affect the ability of the receptor to bind a Tat substrate. Using site-specific cross-linking, we found that the P221R and L225P substitutions did not appear to affect the ability of TatA or TatB to interact with the TMH5 or TMH6 binding sites, although MD simulations suggested that TatA binding at the TMH6 should be partially destabilized by either of these substitutions. At present, the explanation for the inactivity of these variant TatC proteins is unclear. It may be that they are unable to undergo conformational changes associated with substrate transport, but we currently lack biochemical tools to address this further.

In a deliberate attempt to disrupt the TMH6 binding site, we also introduced three bulky amino acid sidechains at positions that contact bound TatA [26]. MD simulations indicated that these three substitutions would be expected to disrupt interaction with TatA, and indeed site-specific crosslinking revealed that very little TatA could be detected in this site. However, unlike the P221R, M222R and L225P substitutions, this bulky substituted TatC variant did not noticeably affect Tat transport activity. It should be noted that TatA is in relatively high abundance in *E. coli* membranes [14, 23], and that the TMH6 binding site sits on the outside of the multimeric receptor complex, in close proximity to the membrane [26]. It is therefore possible that despite the reduced affinity of TatA for this mutated binding site, transient interaction is sufficient to support Tat transport.

## Supplementary Data

Supplementary material 1
